# External validation and comparative performance of Marshall, Rotterdam, and Helsinki computed tomography scores in adult traumatic brain injury

**DOI:** 10.1186/s12873-026-01655-z

**Published:** 2026-06-22

**Authors:** Usame Rakip, Serhat Yıldızhan, İhsan Canbek, Ayşe Ertekin, Mehmet Gazi Boyacı, Serhat Korkmaz, Kazım Hazım Öztürk, Adem Aslan

**Affiliations:** 1 Department of Neurosurgery, Faculty of Medicine, Afyonkarahisar Health Sciences University, Afyonkarahisar, Türkiye; 2 Department of Emergency Medicine, Faculty of Medicine, Afyonkarahisar Health Sciences University, Afyonkarahisar, Türkiye

**Keywords:** Traumatic brain injury, CT scoring systems, External validation, Helsinki CT score, Prognostic models, Mortality prediction

## Abstract

**Purpose:**

Computed tomography (CT)-based scoring systems enable standardized quantification of intracranial pathology following traumatic brain injury (TBI). Despite widespread clinical application, external validation studies comparing the Marshall, Rotterdam, and Helsinki CT scores remain limited, particularly in middle-income settings. This study aimed to externally validate these three CT scoring systems and evaluate their incremental prognostic value beyond clinical variables.

**Methods:**

This retrospective cohort study included adult patients with traumatic brain injury requiring hospitalization beyond 24 h at a tertiary neurosurgical center between 2022 and 2024. Two neurosurgeons independently scored admission CT scans using the Marshall, Rotterdam, and Helsinki CT scoring systems. The primary outcome was in-hospital mortality, and the secondary outcome was poor functional outcome (Glasgow Outcome Scale 1–3) at hospital discharge. Discriminative performance was assessed using area under the receiver operating characteristic curve (AUC) with DeLong test comparisons. Multivariable logistic regression evaluated incremental prognostic value beyond age and admission Glasgow Coma Scale (GCS).

**Results:**

Among 657 patients (mean age 45.9 years, 74.9% male; mild TBI 59.4%, moderate 25.1%, severe 15.5%), in-hospital mortality was 11.0% and poor functional outcome occurred in 23.9%. Among CT-based scores, the Helsinki CT score showed the highest standalone discriminative performance for mortality (AUC 0.793, 95% CI 0.731–0.855), followed by Rotterdam (AUC 0.766, 0.700–0.832) and Marshall (AUC 0.682, 0.625–0.739); Helsinki and Rotterdam both outperformed Marshall. However, when added to a baseline clinical model (age + GCS, AUC 0.876), all CT scores provided minimal to negligible incremental discrimination, with no clinically meaningful improvement in model performance (ΔAUC < 0.005 across all models, all *p* > 0.20). Similar patterns were observed for poor functional outcome.

**Conclusion:**

The Helsinki CT score demonstrated the highest standalone discriminative performance; however, all three CT-based scoring systems provided limited incremental prognostic value beyond clinical assessment (age and GCS), supporting their role as complementary rather than independent tools in TBI evaluation. These findings support the use of CT-based scores primarily for early risk stratification and standardized communication in emergency settings, rather than as standalone prognostic tools.

## Introduction

Traumatic brain injury (TBI) remains a major global public health problem. Affecting nearly 69 million individuals annually, TBI contributes substantially to both mortality and long-term neurological disability [1–4]. Thus, accurate early prognostication is essential for clinical decision-making, resource allocation, family counseling, and research stratification. Computed tomography (CT) provides critical structural information and has long been acknowledged as a cornerstone of TBI assessment. Over the past three decades, several CT-based classification and scoring systems were developed to standardize radiological interpretation and enhance prognostic accuracy.

The Marshall CT Classification, introduced in 1991, was the first widely adopted approach and incorporated cisternal status, degree of midline shift, and presence of mass lesions [5]. Subsequent validation studies have reported area under the receiver operating characteristic (ROC) curve (AUC) generally ranging from 0.55 to 0.70 for mortality prediction [6–8].

The Rotterdam CT score, published in 2005, expanded the Marshall framework by introducing a six-point ordinal scale that incorporates basal cistern compression, midline shift, epidural hematoma, and intraventricular or subarachnoid hemorrhage [6]. External validation studies have demonstrated an AUC consistently between 0.70 and 0.78 [9–11].

More recently, the Helsinki CT score, developed in 2014 as a multidimensional 12-point scoring system, facilitated detailed assessment of traumatic subarachnoid hemorrhage (density and extent), intraventricular hemorrhage, cisternal compression, midline shift, and thalamic or brainstem lesions [12]. Validation studies have reported AUCs ranging from 0.78 to 0.85, suggesting superior discriminative ability relative to earlier models [12–14].

Despite widespread use of these systems, external validation across diverse geographic and clinical settings remains limited [15, 16]. Prognostic performance varies depending on the differences in patient epidemiology, injury mechanisms, prehospital transport systems, and institutional care pathways [10, 17]. Furthermore, no study has conducted direct head-to-head comparisons of all three scoring systems within a single contemporary patient cohort [18].

This study aimed to conduct a comprehensive external validation of the Marshall CT Classification, Rotterdam CT score, and Helsinki CT score in a large tertiary neurosurgical cohort from Türkiye. We hypothesized that the Helsinki CT score would demonstrate the highest standalone discriminative performance among the three systems for mortality and functional outcomes, and we sought to formally evaluate the incremental prognostic value of CT-based scoring systems beyond core clinical variables such as age and admission Glasgow Coma Scale (GCS) score. In emergency care settings, early CT-based risk stratification plays a central role in initial triage, disposition, and resource allocation decisions.

## Methods

### Study design and setting

This retrospective cohort study was conducted between January 2022 and December 2024 at Afyonkarahisar Health Sciences University Hospital, a tertiary neurosurgical referral center in Türkiye that serves multiple provinces with an emergency department. The hospital provides 24-hour neurosurgical coverage and serves urban, suburban, and rural populations. The study was approved by the Afyonkarahisar Health Sciences University Clinical Research Ethics Committee (Approval No: 2026/1, Decision No: 19, dated January 9, 2026) and followed the Transparent Reporting of a multivariable prediction model for Individual Prognosis Or Diagnosis (TRIPOD) guidelines for prognostic model validation studies [19, 20] and was additionally reported in accordance with the STROBE Statement for observational studies (see STROBE checklist).

### Patient selection

The study included consecutive adult patients (≥ 18 years) with traumatic brain injury who underwent admission CT within 24 h of injury and required hospitalization beyond 24 h. All severity strata (mild [GCS 13–15], moderate [GCS 9–12], and severe [GCS 3–8]) were eligible; patients with mild TBI were retained when their admission warranted inpatient observation for intracranial injury, consistent with real-world emergency department triage. The exclusion criteria were as follows: age < 18 years, penetrating TBI, isolated skull fractures without intracranial injury, missing or nondiagnostic CT images, and patients transferred from other hospitals > 24 h after the initial injury. Electronic medical records and institutional trauma registries were systematically reviewed to identify eligible patients.

### Sample size justification

As this retrospective cohort study included all consecutive eligible patients during the study period (2022–2024), the final sample size was 657 patients, representing the total available population. Given the retrospective design, no a priori power calculation was applicable.

### Missing data handling

All key clinical and radiological predictors, as well as outcome variables, were reviewed for completeness. No critical missing data were identified; therefore, analyses were conducted using complete-case data without the need for imputation.

### CT-based scoring systems

Two board-certified neurosurgeons with ≥ 10 years of neurosurgical experience each independently reviewed all admission CT scans and applied the Marshall CT Classification [5], Rotterdam CT score [6], and Helsinki CT score [12] according to their original published definitions. The scorers were blinded to the clinical outcomes. The Marshall CT Classification categorizes TBI into six groups based on cisternal visibility, midline shift, and presence of mass lesions. The Rotterdam CT score ranges from 1 to 6 points, incorporating basal cistern status, midline shift, epidural mass lesion, and presence of subarachnoid or intraventricular blood. The Helsinki CT score ranges from 0 to 12 points, with higher scores indicating more severe injury, and assesses traumatic subarachnoid hemorrhage (density and extent), intraventricular hemorrhage, cisternal compression, midline shift, and thalamic or brainstem lesions.

Inter-rater reliability was assessed using Cohen’s kappa, which demonstrated near-perfect agreement according to established criteria [21]: Marshall κ = 0.89, Rotterdam κ = 0.90, Helsinki κ = 0.88. Discrepancies were resolved by consensus review.

### Outcome assessment

The primary outcome was in-hospital mortality. The secondary outcome was poor functional outcome at hospital discharge, defined as a Glasgow Outcome Scale (GOS) score of 1–3 (death, vegetative state, or severe disability) [22]. To ensure completeness and accuracy, outcome data were extracted from electronic medical records and cross-validated with institutional mortality databases.

### Statistical analysis

Continuous variables were reported as mean ± standard deviation or as median [interquartile range] depending on distribution assessed using the Shapiro–Wilk test. Categorical variables were presented as frequencies and percentages. Baseline characteristics were compared across outcome groups using independent t-tests or Mann–Whitney U tests for continuous variables and chi-square or Fisher’s exact tests for categorical variables.

The discriminative performance of each CT-based scoring system was evaluated by ROC curve analysis. The AUC and 95% CIs were calculated using the DeLong method [23, 24]. Optimal thresholds were identified using the Youden index [25]. Pairwise comparisons of correlated ROC curves were performed using the DeLong test [23], with AUC values interpreted in line with established conventions [26].

To assess the independent prognostic contribution of CT-based scores beyond established clinical predictors, multivariable logistic regression models were constructed. The baseline clinical model included age and admission GCS. Subsequent models added each CT-based scoring system individually. Model discrimination was compared using AUCs with DeLong tests for paired comparisons. Significance was set at *p* < 0.05. All analyses were performed using IBM SPSS Statistics for Windows version 26.0 (IBM Corp, Armonk, NY, USA) and R version 4.2.0 (R Foundation for Statistical Computing, Vienna, Austria).

Given the limited number of outcome events, all multivariable models were deliberately kept parsimonious to minimize the risk of overfitting.

## Results

### Patient characteristics

**Fig. 1:**
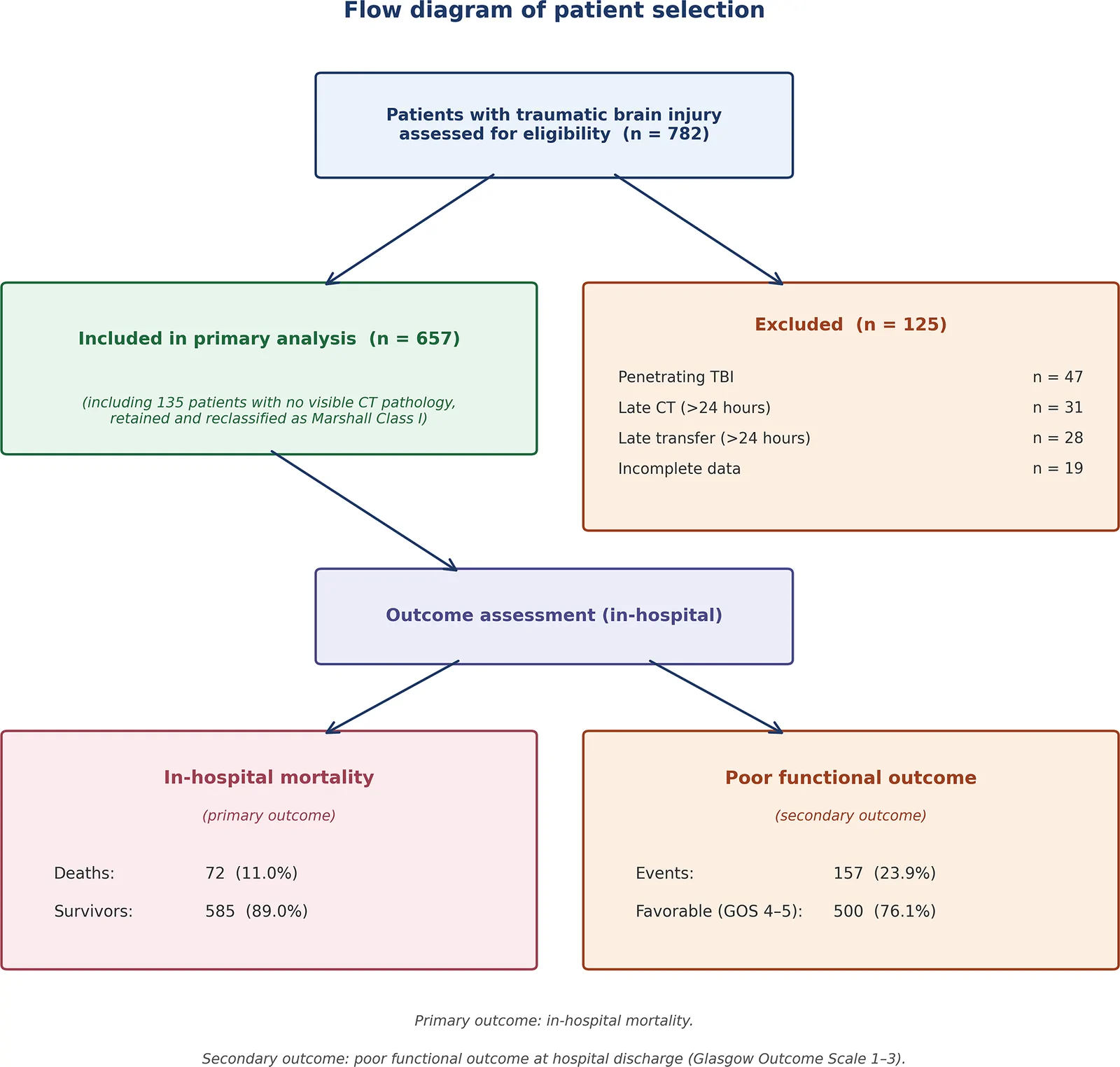
Flow diagram of patient selection. Of 782 patients with traumatic brain injury assessed for eligibility, 125 were excluded (47 penetrating TBI, 31 late CT beyond 24 h, 28 late transfer beyond 24 h, and 19 with incomplete data), yielding 657 patients for the primary analysis. Within the analytic cohort, 135 patients with no visible intracranial pathology on admission CT were retained and reclassified as Marshall Class I, consistent with the original Marshall classification scheme. In-hospital mortality occurred in 72 patients (11.0%) and poor functional outcome (Glasgow Outcome Scale 1–3 at hospital discharge) in 157 patients (23.9%)

Of the 782 patients screened, 125 were excluded (47 penetrating TBI, 31 late CT, 28 late transfer from other hospitals, 19 with incomplete data), leaving 657 patients included in the primary analysis (Fig. 1; Table 1). Of these 657, 135 patients with no visible intracranial pathology on admission CT were retained and reclassified as Marshall Class I in the revised analysis. CT-based score distributions are presented in Table 2A–C. The Rotterdam CT score (Table 2A) demonstrated a mean of 3.05 ± 1.38, with nearly half of the patients scoring 2 points (47.6%). The Marshall CT Classification (Table 2B) was dominated by type IV injuries (32.3%) and type VI (26.0%); 135 patients (20.5%) with no visible intracranial pathology were retained and classified as Marshall Class I, while type II comprised the residual 10 patients (1.5%) after reclassification. The Helsinki CT score (Table 2C) had a mean of 2.77 ± 2.84, with scores distributed across the 0–12 range. The median admission GCS score was 14 (IQR 10–15), with 390 (59.4%) classified as mild, 165 (25.1%) as moderate, and 102 (15.5%) as severe TBI. Injury mechanisms included motor vehicle collisions (33.3%), falls (32.1%), motorcycle collisions (21.5%), and assaults (13.1%). The in-hospital mortality rate was 11.0% (72/657), and the poor functional outcome rate (Glasgow Outcome Scale 1–3 [27]) was 23.9% (157 patients).

**Table 1:** Patient characteristics and baseline demographics (*n* = 657)

Characteristic	Overall (*n* = 657)	Survivors (*n* = 585)	Non-survivors (*n* = 72)	*P* value
**Age**,** years (mean ± SD)**	45.9 ± 17.6	47.1 ± 17.7	36.3 ± 13.4	< 0.001
**Sex**,** n (%)**				0.457
Male	492 (74.9)	435 (74.4)	57 (79.2)	
Female	165 (25.1)	150 (25.6)	15 (20.8)	
**Admission GCS**,** median (IQR)**	14 (10–15)	14 (11–15)	6 (4–10)	< 0.001
**TBI severity**,** n (%)**				< 0.001
Mild (GCS 13–15)	390 (59.4)	387 (66.2)	3 (4.2)	
Moderate (GCS 9–12)	165 (25.1)	141 (24.1)	24 (33.3)	
Severe (GCS 3–8)	102 (15.5)	57 (9.7)	45 (62.5)	
**Injury mechanism**,** n (%)**				< 0.001
Motor vehicle collision	219 (33.3)	196 (33.5)	23 (31.9)	
Fall	211 (32.1)	198 (33.8)	13 (18.1)	
Motorcycle collision	141 (21.5)	112 (19.1)	29 (40.3)	
Assault	86 (13.1)	79 (13.5)	7 (9.7)	

GCS = Glasgow Coma Scale; IQR = interquartile range; TBI = traumatic brain injury. P values from Chi-square test for categorical variables and Mann-Whitney U test for continuous variables. The younger age observed in the non-survivor group reflects a higher prevalence of high-energy motorcycle trauma and severe TBI within this subgroup; see Discussion for interpretation

**Table 2:** Distribution of CT score categories

**A) Rotterdam CT Score (1–6)**		
**Score**	***n***	**%**
1	33	5.0
2	313	47.6
3	45	6.8
4	172	26.2
5	42	6.4
6	52	7.9
**B) Marshall CT Classification (Types I–VI)**		
**Type**	**n**	**%**
I	135	20.5
II	10	1.5
III	61	9.3
IV	212	32.3
V	68	10.4
VI	171	26.0
**C) Helsinki CT Score (0–12)**		
**Score**	**n**	**%**
0	147	22.4
1	143	21.8
2	96	14.6
3	79	12.0
4	52	7.9
5	32	4.9
6	24	3.7
7	19	2.9
8	25	3.8
9	14	2.1
10	14	2.1
11	8	1.2
12	4	0.6

A: Mean ± SD: 3.05 ± 1.38 Median: 2 Range: 1–6

B: Mean ± SD: 3.88 ± 1.77 Median: 4 Range: 1–6. Class I includes 135 patients with no visible intracranial pathology on CT, classified according to the original Marshall scheme

C: Mean ± SD: 2.77 ± 2.84 Median: 2 Range: 0–12

### CT-based score distributions

CT-based score distributions are summarized in Table 2A–C. The Rotterdam CT score averaged 3.05 ± 1.38 (median 2, range 1–6), with nearly half of the patients scoring 2 points (47.6%). The Marshall CT Classification averaged 3.88 ± 1.77 (median 4), dominated by types IV (32.3%) and VI (26.0%). The Helsinki CT score demonstrated the broadest variability (mean 2.77 ± 2.84, median 2, range 0–12). Most patients (70.8%, *n* = 465) scored 0–3 points, whereas 4.0% (*n* = 26) scored 10–12, representing the highest-risk strata.

### Mortality across CT-based score categories

Illustrative clinical thresholds were examined using observed mortality gradients. For the Helsinki CT score, a threshold of ≥ 4 identified the majority of deaths with moderate specificity, whereas ≥ 7 identified a smaller but very high-risk subset with high specificity. Similar patterns were noted for the Rotterdam CT score, where ≥ 3 marked an increasing risk and ≥ 5 identified a high-specificity high-risk group. Among the scoring systems, mortality increased monotonically with higher score categories (Table 3A–C). For the Rotterdam CT score, mortality increased from 3.0% for score 1 to 34.6% for score 6. The Marshall CT Classification demonstrated mortality rates ranging from 2.2% for Class I (no visible pathology) to 29.4% for type V. The Helsinki CT score showed the most pronounced gradient, with 4.9% mortality for scores 0–3, rising sharply to 38.5% for scores 10–12. This pattern demonstrates preserved risk stratification performance in the external cohort.

**Table 3:** In-hospital mortality by CT score category

**A) Rotterdam CT Score**			
**Score**	**Total (n)**	**Deaths (n)**	**Mortality (%)**
1	33	1	3.0
2	313	10	3.2
3	45	6	13.3
4	172	21	12.2
5	42	16	38.1
6	52	18	34.6
**B) Marshall CT Classification**			
**Type**	**Total (n)**	**Deaths (n)**	**Mortality (%)**
I	135	3	2.2
II	10	1	10.0
III	61	6	9.8
IV	212	12	5.7
V	68	20	29.4
VI	171	30	17.5
**C) Helsinki CT Score (grouped)**			
**Score Group**	**Total (n)**	**Deaths (n)**	**Mortality (%)**
0–3	465	23	4.9
4–6	108	18	16.7
7–9	58	21	36.2
10–12	26	10	38.5

A: *p* < 0.001 for trend

B: *p* < 0.001 for trend. Highest mortality in type V (evacuated mass lesion). Class I corresponds to the no-pathology group (*n* = 135; mortality 2.2%)

C: *p* < 0.001 for trend. Clear stepwise increase in mortality with increasing score categories

### External validation: mortality prediction

**Fig. 2:**
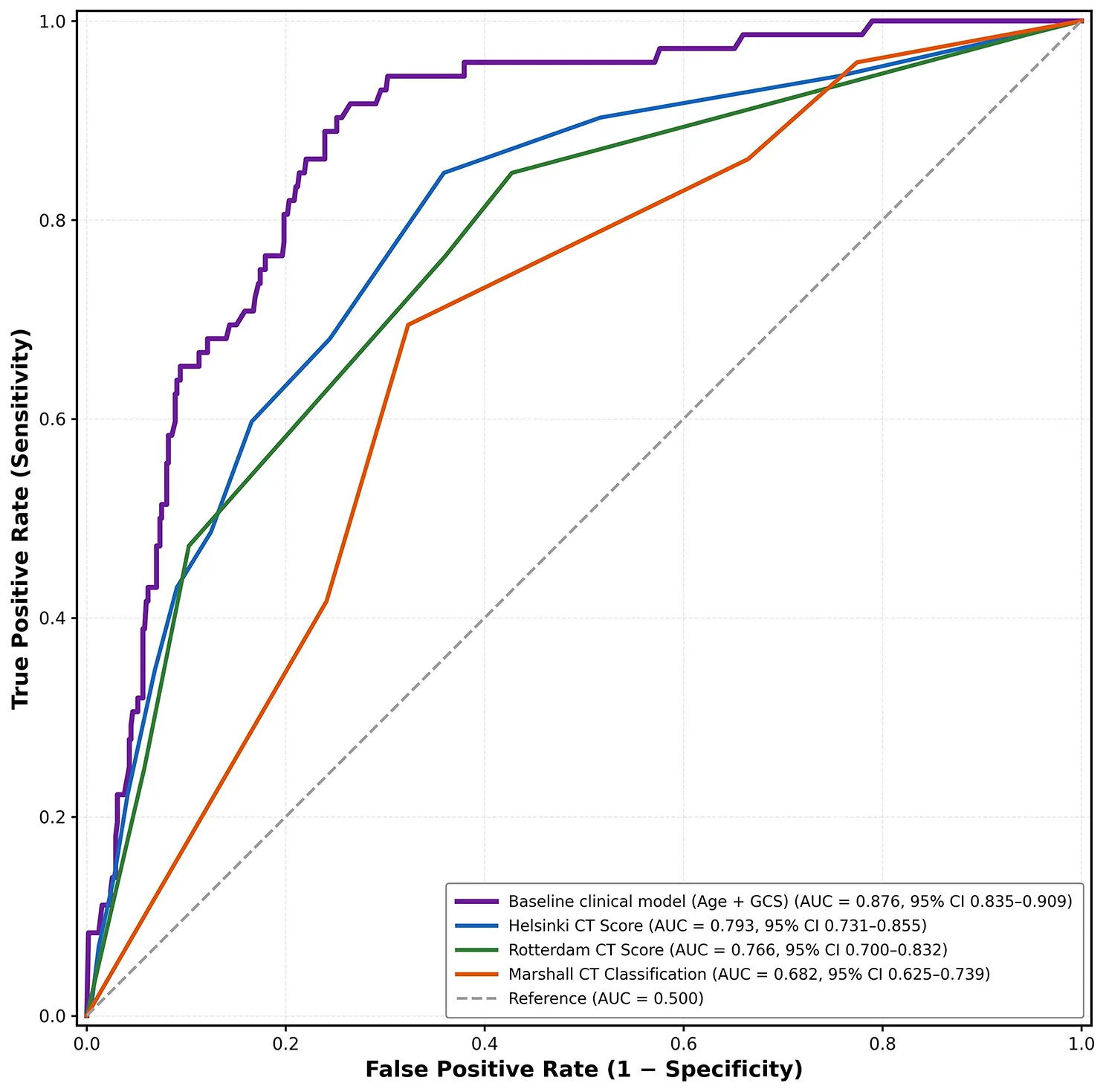
Receiver operating characteristic curves for the prediction of in-hospital mortality, comparing a baseline clinical model (age + admission Glasgow Coma Scale) with three CT-based scoring systems. The baseline clinical model achieved the highest standalone discrimination (AUC = 0.876, 95% CI 0.835–0.909), followed by the Helsinki CT score (AUC = 0.793, 95% CI 0.731–0.855), Rotterdam CT score (AUC = 0.766, 95% CI 0.700–0.832), and Marshall CT Classification (AUC = 0.682, 95% CI 0.625–0.739). Among the CT-based scores, Helsinki and Rotterdam both significantly outperformed Marshall (DeLong test, *p* < 0.001 and *p* = 0.024, respectively); the difference between Helsinki and Rotterdam was not significant (*p* = 0.15). The dashed grey line represents the reference (AUC = 0.500). Baseline clinical model = age + admission GCS

Receiver operating characteristic analyses demonstrated significant discriminative ability for all three scoring systems (Table 4), with corresponding ROC curves shown in Fig. 2. The Helsinki CT score achieved an AUC of 0.793 (95% CI 0.731–0.855), followed by the Rotterdam CT score with an AUC of 0.766 (95% CI 0.700–0.832) and the Marshall CT Classification with 0.682 (95% CI 0.625–0.739).

**Table 4:** Discriminative performance of CT scoring systems

**Scoring System**		**In-Hospital Mortality AUC (95% CI)**		**Poor Functional Outcome AUC (95% CI)**	
Helsinki CT Score		0.793 (0.731–0.855)		0.726 (0.672–0.780)	
Rotterdam CT Score		0.766 (0.700–0.832)		0.719 (0.664–0.774)	
Marshall CT Classification		0.682 (0.625–0.739)		0.645 (0.587–0.703)	
**B. Subgroup analysis by TBI severity (mortality)**					
**TBI Severity Subgroup**	**Helsinki AUC (95% CI)**		**Rotterdam AUC (95% CI)**		**Marshall AUC (95% CI)**
Overall cohort (*n* = 657)	0.79 (0.73–0.85)		0.77 (0.70–0.83)		0.68 (0.61–0.75)
Moderate TBI (*n* = 165)	0.41 (0.27–0.55)		0.53 (0.39–0.67)		0.40 (0.26–0.54)
Severe TBI (*n* = 102)	0.50 (0.38–0.62)		0.45 (0.33–0.57)		0.51 (0.39–0.63)

AUC = area under the ROC curve; CI = confidence interval (DeLong method); CT = computed tomography. Poor functional outcome defined as Glasgow Outcome Scale 1–3 at hospital discharge. Pairwise DeLong comparisons (mortality): Helsinki vs. Marshall *p* < 0.001; Rotterdam vs. Marshall *p* = 0.024; Helsinki vs. Rotterdam *p* = 0.15

B: AUC = area under the ROC curve; CI = confidence interval; TBI = traumatic brain injury. Mild TBI subgroup (*n* = 390) not shown due to insufficient mortality events (*n* = 3). The reduced discrimination within the moderate and severe subgroups likely reflects restricted score variability and ceiling effects within these more homogeneous severity strata

Pairwise comparisons revealed significant differences. The Helsinki CT score significantly outperformed the Marshall CT Classification (*p* < 0.001), as did the Rotterdam CT score (*p* = 0.024). The difference between the Helsinki and Rotterdam CT scores was not significant (*p* = 0.15). These results are consistent with the original development cohorts, supporting external validity.

### Functional outcome prediction

**Fig. 3:**
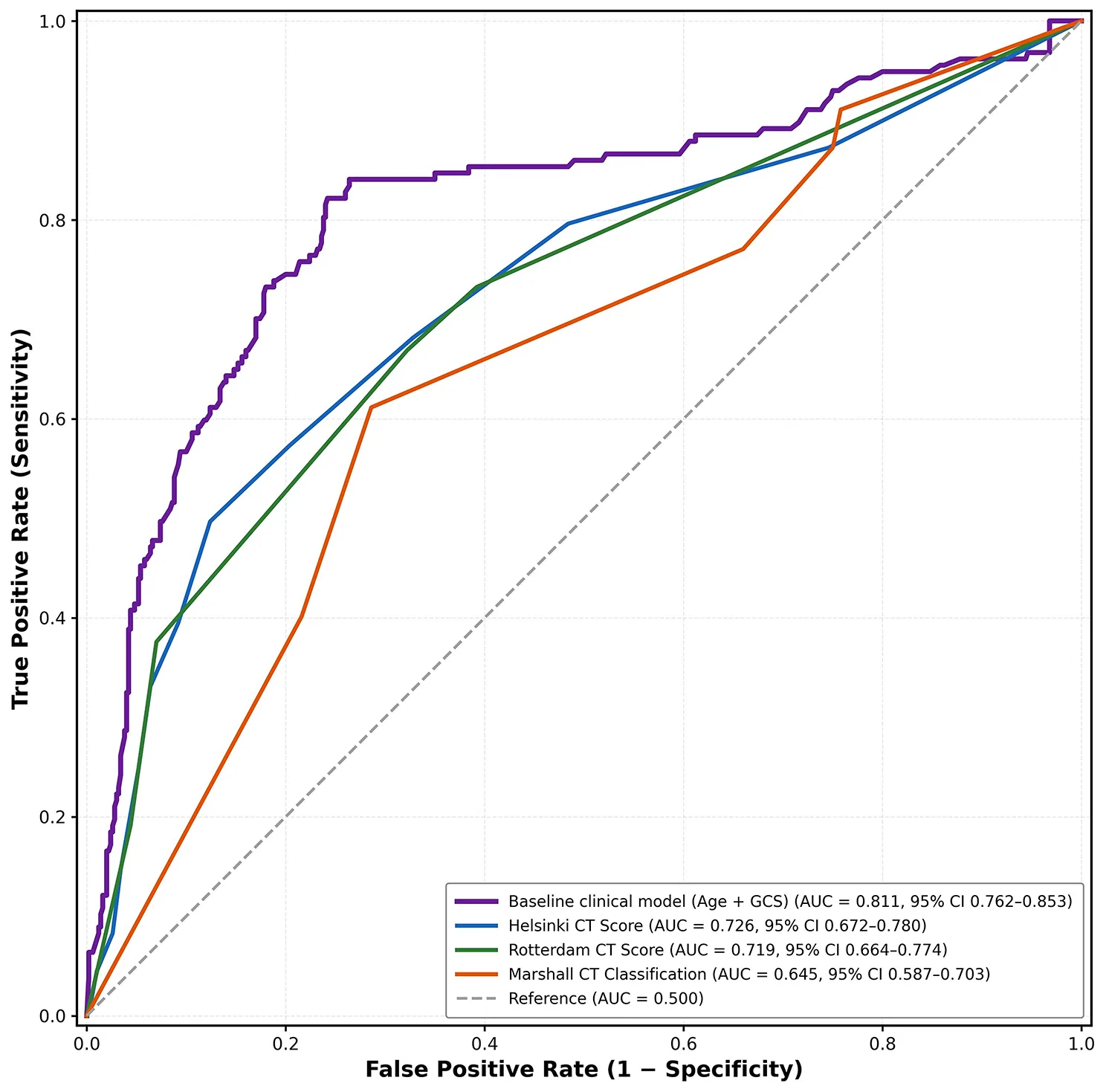
Receiver operating characteristic curves for the prediction of poor functional outcome (Glasgow Outcome Scale 1–3) at hospital discharge, comparing a baseline clinical model (age + admission Glasgow Coma Scale) with three CT-based scoring systems. The baseline clinical model achieved the highest standalone discrimination (AUC = 0.811, 95% CI 0.762–0.853), followed by the Helsinki CT score (AUC = 0.726, 95% CI 0.672–0.780), Rotterdam CT score (AUC = 0.719, 95% CI 0.664–0.774), and Marshall CT Classification (AUC = 0.645, 95% CI 0.587–0.703). The dashed grey line represents the reference (AUC = 0.500). Baseline clinical model = age + admission GCS

For the prediction of poor functional outcome (GOS 1–3), the Helsinki CT score again performed best, achieving an AUC of 0.726 (95% CI 0.672–0.780). The Rotterdam CT score yielded an AUC of 0.719 (95% CI 0.664–0.774), and the Marshall CT Classification demonstrated 0.645 (95% CI 0.587–0.703). ROC curves for functional outcomes are presented in Fig. 3.

Differences were significant between both the Helsinki CT score and Marshall CT Classification (*p* = 0.021) and between the Rotterdam CT score and Marshall CT Classification (*p* = 0.034). Rotterdam and Helsinki CT scores showed comparable performance (*p* = 0.336).

### Incremental value beyond clinical variables

When each CT-based score was added to a baseline multivariable model including age and admission GCS, all three systems provided minimal to negligible incremental discrimination. For mortality, the baseline clinical model achieved an AUC of 0.876 (95% CI 0.835–0.909); the addition of the Helsinki, Rotterdam, or Marshall CT scores improved discrimination by ΔAUC < 0.005 across all models, with no CT score reaching statistical significance in the multivariable models (Helsinki OR 0.99, 95% CI 0.87–1.12, *p* = 0.84; Rotterdam OR 1.00, 0.77–1.31, *p* = 0.98; Marshall OR 1.03, 0.84–1.28, *p* = 0.76) and DeLong comparisons showing no clinically meaningful gain (all *p* > 0.20) (Table 5). For poor functional outcome, the baseline clinical model achieved an AUC of 0.811 (95% CI 0.762–0.853), and the incremental prognostic value of each CT score remained comparably limited (ΔAUC ≤ 0.004; Helsinki OR 0.92, 0.82–1.02, *p* = 0.098; Rotterdam OR 0.97, 0.79–1.18, *p* = 0.75; Marshall OR 0.97, 0.84–1.11, *p* = 0.62) (Table 6).

**Table 5:** Multivariable logistic regression models for in-hospital mortality

Predictor	Model 1: Baseline (Age + GCS)	Model 2: + Marshall	Model 3: + Rotterdam	Model 4: + Helsinki
Age (per year), OR (95% CI), p	0.98 (0.96–0.99), 0.009	0.98 (0.96–0.99), 0.009	0.98 (0.96–0.99), 0.009	0.98 (0.96–0.99), 0.009
GCS (per point), OR (95% CI), p	0.73 (0.68–0.78), < 0.001	0.73 (0.68–0.79), < 0.001	0.73 (0.66–0.80), < 0.001	0.72 (0.65–0.80), < 0.001
Marshall (per category), OR (95% CI), p	—	1.03 (0.84–1.28), 0.76	—	—
Rotterdam (per point), OR (95% CI), p	—	—	1.00 (0.77–1.31), 0.98	—
Helsinki (per point), OR (95% CI), p	—	—	—	0.99 (0.87–1.12), 0.84
**AUC (95% CI)**	0.876 (0.835–0.909)	0.877 (0.837–0.909)	0.876 (0.835–0.910)	0.876 (0.835–0.910)
**ΔAUC vs. baseline**	—	+ 0.002	+ 0.000	+ 0.001
**DeLong p-value vs. baseline**	—	0.22	0.67	0.35

OR = odds ratio; CI = confidence interval; GCS = Glasgow Coma Scale; AUC = area under the ROC curve. ΔAUC values are calculated from unrounded model AUC estimates and may not equal the difference of rounded AUC values shown. CT score odds ratios approach unity, and all multivariable models yielded minimal to negligible incremental discrimination (ΔAUC < 0.005 across all models). The inverse association observed for age in this cohort reflects differential injury mechanism distribution (younger patients disproportionately affected by high-energy motorcycle collisions) and should be interpreted with caution and in the context of cohort-specific injury patterns; see Discussion

**Table 6:** Multivariable logistic regression models for poor functional outcome

Predictor	Model 1: Baseline (Age + GCS)	Model 2: + Marshall	Model 3: + Rotterdam	Model 4: + Helsinki
Age (per year), OR (95% CI), p	0.99 (0.98–1.01), 0.39	0.99 (0.98–1.01), 0.39	0.99 (0.98–1.01), 0.39	0.99 (0.98–1.01), 0.36
GCS (per point), OR (95% CI), p	0.72 (0.68–0.76), < 0.001	0.71 (0.67–0.76), < 0.001	0.71 (0.66–0.77), < 0.001	0.68 (0.62–0.74), < 0.001
Marshall (per category), OR (95% CI), p	—	0.97 (0.84–1.11), 0.62	—	—
Rotterdam (per point), OR (95% CI), p	—	—	0.97 (0.79–1.18), 0.75	—
Helsinki (per point), OR (95% CI), p	—	—	—	0.92 (0.82–1.02), 0.098
**AUC (95% CI)**	0.811 (0.762–0.853)	0.811 (0.762–0.853)	0.812 (0.762–0.853)	0.815 (0.766–0.856)
**ΔAUC vs. baseline**	—	+ 0.000	+ 0.001	+ 0.004
**DeLong p-value vs. baseline**	—	0.85	0.42	0.30

OR = odds ratio; CI = confidence interval; GCS = Glasgow Coma Scale; AUC = area under the ROC curve. Poor functional outcome defined as Glasgow Outcome Scale 1–3 at hospital discharge. CT-based scores provided minimal to negligible incremental discrimination beyond the baseline clinical model (ΔAUC ≤ 0.004 across all models)

### Subgroup analysis by TBI severity

Subgroup analysis (Table 4B) revealed diminished discriminative performance within the moderate and severe TBI strata. In the overall cohort, AUCs were 0.79, 0.77, and 0.68 for the Helsinki, Rotterdam, and Marshall CT scoring systems, respectively. However, within the moderate TBI subgroup (*n* = 165), AUCs ranged from 0.40 to 0.53 across the three systems. Similarly, in the severe TBI subgroup (*n* = 102), AUCs ranged from 0.45 to 0.51. The attenuated AUCs reflect restricted score variability and ceiling effects within these homogeneous severity groups.

## Discussion

### Principal findings and external validity

To the best of our knowledge, this study provides the first comprehensive head-to-head external validation of the Marshall, Rotterdam, and Helsinki CT scoring systems in a contemporary tertiary neurosurgical cohort from Türkiye. In this population, the Helsinki CT score demonstrated the highest discriminative ability for both in-hospital mortality (AUC 0.793) and poor functional outcome (AUC 0.726), with Rotterdam demonstrating intermediate performance and the Marshall CT Classification performing least well (AUCs 0.682 and 0.645, respectively). Notably, despite differences in population demographics, trauma mechanisms, and healthcare systems, the AUCs in our cohort closely paralleled those reported in the original derivation studies from the United States, the Netherlands, and Finland [5, 6, 12]. This concordance supports the external validity of these CT-based prognostic models and indicates that their performance is largely maintained in our geographic and clinical context. From an emergency medicine perspective, these findings highlight the role of CT-based scores in early triage decisions, particularly when clinical information may be limited at initial presentation.

### Comparison with previous literature

Our results support pooled estimates from previous validation studies and meta-analyses. The Marshall CT Classification again demonstrated modest prognostic performance (AUC 0.68), in line with the frequently reported range of 0.55–0.70 [6–8]. The Rotterdam CT score performed within its expected AUC range of approximately 0.70–0.78, similar to European and North American cohorts [6, 9–11]. The Helsinki CT score reproduced its originally reported higher performance (AUC 0.78–0.85) [12–14]. This finding is valuable given substantial differences in trauma epidemiology and prehospital systems between Scandinavian centers and our setting. The consistency of these findings across healthcare environments indicates that the Helsinki CT scoring system may represent a relatively stable and transportable prognostic tool.

The comparatively better performance of the Helsinki CT score likely reflects its broader inclusion of radiological prognostic markers. Compared with the more categorical Marshall CT system, the Helsinki CT score incorporates detailed assessment of traumatic subarachnoid hemorrhage (including density and distribution), intraventricular hemorrhage, midline shift severity, cisternal compression, and thalamic or brainstem lesions [12]. Each component is associated with outcome in multivariable analyses, and their integration into a composite score may increase prognostic precision [7].

### Clinical versus radiological prognostication

This multivariable analysis emphasizes the dominant contribution of core clinical variables, particularly age and admission GCS, to in-hospital mortality prediction. Despite robust univariable discrimination (Helsinki AUC 0.793, Rotterdam AUC 0.766, and Marshall AUC 0.682), none of these scoring systems remained significant when added to models that already included age and GCS. The model AUCs did not change (0.876–0.877), indicating that CT-based scoring systems demonstrated limited incremental prognostic value beyond these clinical predictors. This pattern supports the findings from major studies on TBI prognostic model development.

In the IMPACT study, the core model based on age, GCS motor score, and pupillary reactivity yielded an AUC of approximately 0.80 for 6-month mortality and unfavorable outcomes, with CT characteristics adding only small incremental prognostic value (AUC + 0.02–0.03) [28]. Similarly, in the CRASH model, CT variables contributed only modest incremental prognostic value beyond baseline clinical severity, particularly for short-term mortality [28, 29]. These findings are biologically plausible, as more severe clinical presentations, such as lower GCS scores, generally reflect more extensive structural brain injury, resulting in a substantial overlap between clinical and radiological domains.

These findings should not be taken to undermine the clinical usefulness of CT-based scoring systems. Their strong univariable performance demonstrates their utility in situations where clinical data are incomplete, unreliable, or delayed, such as during interfacility transfer, in a prehospital environment, or in resource-limited settings. Furthermore, CT-based scores summarize critical anatomical features that are essential to neurosurgical planning, decisions regarding intracranial pressure monitoring, and standardized communication among teams [16, 17, 30]. Specifically, the Helsinki CT score offers granular characterization of subarachnoid and intraventricular hemorrhage and mass effect, which can inform surgical and critical care strategies even when they add only limited value to formal mortality models. In this context, CT-based scores may be particularly useful for early triage, prognostic communication, and risk enrichment rather than as standalone tools for outcome prediction.

The strong independent effect of admission GCS on our models reinforces its importance as a frontline prognostic tool [18]. GCS is immediately available and reproducible and does not depend on imaging resources, making it particularly valuable in emergency and prehospital settings. Nevertheless, integrating clinical and radiological information remains crucial for comprehensive assessment, and CT-based scoring systems continue to play a key role in risk stratification, research standardization, and clinical decision-making.

A key observation from our analysis is the divergence between standalone and multivariable performance of CT-based scoring systems. While the Helsinki score demonstrated the highest standalone discrimination among CT scores (AUC 0.793 for mortality), this advantage disappeared after adjustment for age and GCS, highlighting the dominant prognostic role of clinical variables. In multivariable models, the addition of any CT score to age and GCS improved discrimination by less than 0.005 AUC units, representing no clinically meaningful gain in predictive performance (all *p* > 0.20), with CT score odds ratios approaching unity (Helsinki OR 0.99, 95% CI 0.87–1.12, *p* = 0.84).

Unlike some previous reports where Helsinki demonstrated statistically significant superiority over Rotterdam, the difference between these two scores was not statistically significant in our cohort (AUC 0.793 vs. 0.766 for mortality, *p* = 0.15). This may reflect differences in case-mix, injury severity distribution, or sample size, and is consistent with the overlapping confidence intervals observed in our analysis. Both Helsinki and Rotterdam nonetheless substantially outperformed Marshall (*p* < 0.001 and *p* = 0.024, respectively).

Our findings should be interpreted in the context of large-scale prognostic studies incorporating the full IMPACT core variable set. In IMPACT and CRASH external validations, the addition of CT characteristics to core clinical predictors (age, motor GCS, pupillary reactivity) improved discrimination by approximately 0.02–0.03 AUC units [28, 29]. The incremental prognostic value of CT scores in our cohort (ΔAUC < 0.005) was even smaller, likely reflecting the strong discrimination of our baseline clinical model (AUC 0.876) — a ceiling effect that warrants consideration — but nonetheless underscores that the added value of CT-based scoring beyond core clinical assessment remains modest across diverse cohorts and clinical settings.

The observed protective association between age and mortality in our multivariable models (OR 0.98 per year, *p* = 0.009) appears counterintuitive relative to established TBI literature, where advanced age is consistently a predictor of worse outcome. This pattern reflects the distinctive case-mix of our cohort: younger patients were disproportionately affected by high-energy mechanisms such as motorcycle collisions (mortality 20.6%), while older patients predominantly sustained lower-energy falls (mortality 6.2%). Although this pattern remained after adjustment for available covariates (including trauma mechanism and TBI severity), it likely reflects residual confounding and cohort-specific case-mix. Accordingly, the age coefficient in our models should be interpreted as likely reflecting cohort-specific confounding rather than a universal protective effect of age in TBI.

The clinical utility of CT-based scoring systems likely varies across healthcare settings. In tertiary neurosurgical centers where subspecialty radiological expertise is readily available, the granular detail of Helsinki CT scoring (including subarachnoid hemorrhage density quantification, ventricular assessment, and brainstem evaluation) may be feasibly applied and provide clinically meaningful prognostic refinement. In contrast, in central or regional hospitals where CT interpretation may be performed by emergency physicians or general radiologists, simpler scoring systems such as Rotterdam or clinical variables alone (age, admission GCS) may offer a more pragmatic balance between accuracy and implementation feasibility. Future implementation studies should address CT score interpretability and inter-observer reliability across these varied clinical contexts.

### Limitations of the Marshall CT classification

Despite its long-standing use, the Marshall CT Classification demonstrated the weakest discrimination in our cohort, which supports much of the modern literature. Several factors may explain this finding. First, binary radiological variables (e.g., cisterns present vs. absent) may obscure clinically meaningful gradations of injury severity [6]. Second, the absence of explicit assessment of subarachnoid and intraventricular hemorrhage omits two established prognostic indicators [7, 12, 31]. Third, the scoring system was developed in an earlier era of CT technology and neurosurgical practice [32], including before the widespread adoption of decompressive craniectomy, which may reduce its relevance in contemporary care pathways [33, 34].

Practice variations in TBI management across centers [35] may influence the external validity of our findings. Nevertheless, the Marshall CT Classification remains useful in many settings because of its simplicity, familiarity, and continued use as a common language in clinical communication and triage.

### Clinical implications

Exploratory threshold findings further highlight the potential clinical utility of CT-based scoring systems. For instance, a Helsinki CT score of ≥ 4 identified most mortality events with moderate specificity, whereas a score of ≥ 7 delineated a smaller group with substantially higher risk. These thresholds, derived directly from observed score–mortality gradients, may practically guide early triage and counseling. These threshold-based findings should be interpreted as descriptive and hypothesis-generating rather than as statistically optimized decision limits. Several practical implications emerge from these findings. In this cohort, the Helsinki CT score demonstrated the strongest discriminative performance for mortality and functional outcome; thus, it may be a suitable choice when a single CT-based scoring system is desired for risk stratification. Its monotonic association with mortality across score ranges allows for intuitive interpretation during discussions with families and multidisciplinary care teams. For research applications, the greater granularity of the Helsinki CT score may improve case-mix adjustment and reduce residual confounding in clinical trials [18, 36].

Although the Helsinki CT scoring system is somewhat more time-consuming than simpler classification schemes, our high inter-rater reliability (κ = 0.88) indicates that consistent application is feasible with limited training. The future development of semiautomated or fully automated CT-based scoring systems integrated into the picture archiving and communication system or electronic medical record systems may further enhance feasibility and promote adoption in routine practice. Given the substantial healthcare costs associated with severe TBI, even modest improvements in early risk stratification may have meaningful economic implications [37].

### Strengths and limitations

This study has several strengths. First, to the best of our knowledge, it represents the largest external validation of these CT-based scoring systems conducted in Türkiye. Second, the inclusion of consecutive patients reduced the likelihood of selection bias, and the overall sample size (*n* = 657) allowed stable estimation of AUCs. Third, blinded dual scoring by experienced neurosurgeons, along with excellent inter-rater reliability (all κ ≥ 0.88), supports the robustness of the radiological assessments. Fourth, direct comparison of three major CT-based scoring systems using identical methodology within a single cohort enabled fair comparative evaluation, and the inclusion of mortality and functional outcomes provided a more comprehensive view of prognostic performance. Finally, the incorporation of multivariable models allowed the assessment of the independent contribution of CT-based scores beyond core clinical variables.

Several limitations warrant consideration. First, pupillary reactivity and individual GCS components — variables included in established prognostic models such as IMPACT and CRASH — were not available in our retrospective dataset, reflecting the scope of systematic documentation in our institution’s records during the study period. The absence of these variables may represent a conservative estimate of CT scores’ incremental prognostic value, as their inclusion in the baseline clinical model might have further reduced the marginal contribution of CT-based scoring. Second, although based in a large tertiary hospital with a multi-provincial catchment area, this single-center design limits the generalizability of our findings. Third, the retrospective nature introduces potential for incomplete documentation and outcome misclassification, although we tried to mitigate this through systematic electronic record review and cross-validation with institutional mortality databases. Fourth, outcomes were restricted to in-hospital mortality and discharge GOS, and longer-term outcomes at 6 or 12 months were unavailable [27]. Fifth, calibration metrics, such as the calibration slope or Brier score, were not evaluated, which are relevant for full prognostic model assessment [38, 39], nor was formal recalibration of any scoring system to our local population. These constraints are inherent in retrospective validation studies and should be considered when interpreting the findings. Additionally, temporal changes in trauma care practices and advances in CT imaging technology over the study period may have influenced outcome patterns. These limitations motivate our ongoing efforts to develop a prospective TBI registry that systematically captures all IMPACT-aligned core variables.

### Future research

Future validation in surgical cohorts, including patients undergoing decompressive craniectomy [40], would strengthen the generalizability of these findings across diverse treatment paradigms. Future research could build on these results in several directions. First, multicenter external validation across additional regions, including South Asia, Africa, Eastern Europe, and Latin America, would enable a more comprehensive evaluation of generalizability. Prospective studies employing standardized CT acquisition and blinded scoring may minimize bias and support more detailed calibration analyses. Integrating CT scoring systems with established clinical models, such as IMPACT and CRASH, may further enhance prognostic accuracy by leveraging complementary domains [10, 28, 29].

In addition, machine-learning approaches that incorporate quantitative CT metrics (e.g., lesion volumes and radiomics features) may offer opportunities to develop next-generation prognostic tools [33, 36]. Finally, implementation-focused research on barriers to adoption and testing automated scoring workflows may facilitate the translation of evidence-based CT scoring systems into daily practice.

## Conclusions

In this tertiary neurosurgical cohort, the Helsinki CT score demonstrated the highest standalone discriminative performance among the three widely used CT-based scoring systems for predicting in-hospital mortality and poor functional outcome after TBI, whereas the Rotterdam score showed intermediate and the Marshall CT Classification demonstrated lower performance. However, multivariable analyses demonstrated that age and admission GCS were the strongest independent predictors of outcome, while all three CT-based scoring systems provided limited incremental prognostic value once these clinical factors were included (ΔAUC < 0.005 across all models). This pattern is consistent with major international prognostic models, such as IMPACT and CRASH [28, 29], and highlights the pivotal role of core clinical variables in predicting TBI outcomes. Overall, these findings support the use of CT-based scoring systems, particularly the Helsinki CT score, as complementary rather than independent tools within a broader clinical and radiological assessment framework, while underscoring the need for further multicenter validation and refined integration of clinical and imaging predictors in future prognostic modeling efforts.

## Data Availability

The datasets generated and analyzed during the current study are available from the corresponding author on reasonable request.
